# Over-expressed long noncoding RNA HOXA11-AS promotes cell cycle progression and metastasis in gastric cancer

**DOI:** 10.1186/s12943-017-0651-6

**Published:** 2017-04-26

**Authors:** Zhili Liu, Zhenyao Chen, Ruihua Fan, Bin Jiang, Xin Chen, Qinnan Chen, Fengqi Nie, Kaihua Lu, Ming Sun

**Affiliations:** 1 Department of Oncology, The Affiliated Jiangyin Hospital of Southeast University Medical College, Wuxi, People’s Republic of China; 2 Department of Oncology, Second Affiliated Hospital, Nanjing Medical University, Nanjing, 210011 People’s Republic of China; 3 Department of Medical Oncology, Huai’an First People’s Hospital, Nanjing Medical University, Huai’an, People’s Republic of China; 4 Department of Urology, The Affiliated Jiangyin Hospital of Southeast University Medical College, Wuxi, People’s Republic of China; 5 Department of Oncology, First Affiliated Hospital, Nanjing Medical University, Nanjing, People’s Republic of China; 6 Department of Bioinformatics and Computational Biology, UT MD Anderson Cancer Center, 1400 Pressler Street, Unit 1410, Houston, TX 77030 USA

**Keywords:** Gastric cancer, lncRNA, HOXA11-AS, Cell cycle progression, Metastasis

## Abstract

**Background:**

Long noncoding RNAs (lncRNAs) have emerged as critical regulators in a variety of human cancers, including gastric cancer (GC). However, the function and mechanisms responsible for these molecules in GC are not fully understood. In our previous study, we found that GC associated lncRNA HOXA11-AS is significantly upregulated in GC tissues. Over-expressed HOXA11-AS promotes GC cells proliferation and invasion through scaffolding the chromatin modification factors PRC2, LSD1 and DNMT1.

**Methods:**

HOXA11-AS expression levels in GC cells was detected by quantitative real-time PCR (qPCR). HOXA11-AS siRNAs and overexpression vector were transfected into GC cells to down-regulate or up-regulate HOXA11-AS expression. In vitro and in vivo assays were performed to investigate the functional role of HOXA11-AS in GC cells cell cycle progression, invasion and metastasis. RIP and ChIP assays were used to determine the mechanism of HOXA11-AS’s regulation of underlying targets.

**Results:**

We found that knockdown of HOXA11-AS induced GC cells G0/G1 phase arrest and suppressed GC cells migration, invasion and metastasis in vivo. Moreover, mechanistic investigation showed that HOXA11-AS could interact with WDR5 and promote β-catenin transcription, bind with EZH2 and repress P21 transcription, and induce KLF2 mRNA degradation via interacting with STAU1.

**Conclusions:**

Taken together, these findings show that HOXA11-AS not only could promote GC cells migration and invasion in vitro, but also promotes GC cells metastasis in vivo, at least in part, by regulating β-catenin and KLF2.

**Electronic supplementary material:**

The online version of this article (doi:10.1186/s12943-017-0651-6) contains supplementary material, which is available to authorized users.

## Background

Gastric cancer is one of the most common malignancies and leading causes of cancer-related death worldwide [1, 2]. In spite of the fast improvement of diagnostic, surgical techniques, and discovery of new molecular targeted drug, the 5-years overall survival rate of GC patients is still unsatisfactory [3]. Although more and more oncogenes, tumor suppressors and tumor driver associated mutations have been discovered in the past decades, the molecular mechanisms underlying GC carcinogenesis and progression are still not well documented [4]. Recently, increasing evidence has highlighted the important roles of noncoding RNAs, especially long noncoding RNAs (lncRNAs) in the pathogenesis of multiple human cancers [5–7]. However, little is known about these lncRNAs and their involvement in human GC development and progression.

Over the past years, the advance on the next-generation sequencing technique and bioinformatics methods has lead to the completion of many large-scale and multi-tissue sequencing programs, such as The Encyclopedia of DNA Elements (ENCODE) [8] and The Cancer Genome Atlas (TCGA) [9]. As a result, annotation of these data revealed that only less than 3% of the whole human genome are protein coding genes, while the majority yields thousands of noncoding transcripts [10]. Among these noncoding RNAs, lncRNAs are an newly identified regulatory RNA member with length greater than 200 nucleotides, and no protein coding capacities. A lot of studies have revealed that lncRNAs participate in a wide range of biological processes, and their dysregulation involves in a variety of human diseases [11]. Additionally, lots of cancer-associated lncRNAs have been characterized, and their biological function and underlying molecular mechanisms involved in tumorigenesis have been demonstrated [12]. For example, lncRNA SNHG5 is upregulated in colorectal cancer and promotes cell survival by counteracting STAU1-mediated mRNA destabilization [13].

In the case of GC, TCGA sequencing and several microarray analyses have revealed that sets of lncRNAs expression are misregulated, and our previous studies found that overexpressed lncRNAs HOTAIR [14, 15], HOXA-AS2 [16], DUXAP8 [17] and ZFAS1 [18] exhibit oncogenic function, while MEG3 [19] and GAS5 [20] act as tumor suppressors. In recent study, we identified an new GC associated lncRNA HOXA11-AS, which is significantly upregulated in GC and promotes GC cells proliferation and invasion through scaffolding the chromatin modification factors PRC2, LSD1 and DNMT1 [21]. Here, we further investigated the effect of HOXA11-AS overexpression on GC cells cell cycle progression and in vivo metastasis, and the underlying mechanisms.

## Methods

### Cell lines culture

BGC823, SGC7901 and AGS cell lines were purchased from the Shanghai Cell Bank of the Chinese Academy of Sciences (Shanghai, China). BGC823 cells were cultured in RPMI 1640 (Invitrogen, Shanghai, China); SGC7901 and AGS were cultured in Dulbecco’s modified Eagle’s medium with 10% fetal bovine serum (Invitrogen, Carlsbad, CA, USA). All cell lines were characterized by DNA fingerprinting using short tandem repeat markers from the Shanghai Cell Bank.

### RNA extraction and qRT-PCR assays

Total RNA from GC cells was isolated using TRIzol reagent (Invitrogen) according to the manufacturer’s instructions. 1 μg RNA was reverse-transcribed in a final volume of 20 μl under the standard conditions using the PrimeScript RT reagent kit (Takara, Dalian, China). SYBR Premix Ex Taq (Takara, Dalian, China) was used for the real-time qPCR assays, which were performed on an Applied Biosystems 7500 Real-Time PCR System (Applied Biosystems). The primers sequences used are shown in previous study and Additional file 1: Table S1 [21]. qRT-PCR results were analyzed relative to the threshold cycle (CT) values and then converted to fold changes. GAPDH was used as control.

### siRNA and plasmid vector transfection

Plasmid vectors (pCDNA-HOXA11-AS, sh-HOXA11-AS and empty vectors) for transfection were prepared using DNA Midiprep kits (Qiagen, Hilden, Germany), and then transfected into BGC823,SGC7901, or AGS cells using lipofectamine 3000. si-HOXA11-AS, si-EZH2, si-STAU1, si-WDR5 or negative control were transfected into BGC823 and SGC7901 cells using RNAiMax, All siRNA and shRNA sequences are shown in Additional file 1: Table S1. GC cells were grown in six-well plates and transfected according to the manufacturer’s instructions. At 48 h post-transfection, cells were harvested for qRT-PCR or western blot analyses.

### Cell proliferation assays

Cell proliferation ability was examined using a Cell Counting Kit-8 (Dojindo Molecular Technologies, Inc). For cell cycle analysis, BGC823 and SGC7901 cells were harvested 48 h after transfection by trypsin, and stained with PI using the CycleTEST™ PLUS DNA Reagent Kit (BD Biosciences) following the protocol and analyzed by FACScan. The percentage of the cells in G0/G1, S, and G2/M phase were counted and compared.

### In vivo tumor metastasis assay

Four-week-old female athymic BALB/c nude mice were maintained under specific pathogen-free conditions and manipulated according to the protocols approved by the Shanghai Medical Experimental Animal Care Commission. sh-HOXA11-AS or empty vector stably-transfected BGC823 cells were harvested. Then, 1x10^7^ cells were injected subcutaneously into each mouse tail vein. 7 weeks after injection, the mice were sacrificed, and all the lungs are removed and stained by Hematoxylin and Eosin (HE) Staining. This study was carried out in strict accordance with the recommendations in the Guide for the Care and Use of Laboratory Animals of the National Institutes of Health. The protocol was approved by the Committee on the Ethics of Animal Experiments of Nanjing Medical University.

### RNA and Chromatin immunoprecipitation assasy

The detail of RNA and Chromatin immunoprecipitation assasy has been described in the previous study [21].

### Western blotting and antibodies

BGC823 and SGC7901 cells were lysed with RIPA extraction reagent (Beyotime, Beijing, China) supplemented with a protease inhibitor cocktail (Roche, CA, USA). Proteins (40 μg) were separated by 4–10% sodium dodecyl sulfate polyacrylamide gel electrophoresis, transferred to 0.22 μm polyvinylidene fluoride membranes (Millipore), and then incubated with E-cadherin (cell signaling technologies), N-cadherin, Vimentin, β-catenin, KLF2, P21 or GAPDH (cell signaling technologies) antibodies. ECL chromogenic substrate was used for protein visualization, and the proteins were quantified by densitometry (Quantity One software; Bio-Rad).

### Statistical analysis

Student’s *t*-test (2 tailed), one-way analysis of variance, and the Mann–Whitney *U* test were conducted to analyze the in vitro and in vivo data by SPSS 17.0 software (IBM, IL, USA). *P* values less than 0.05 were considered significant.

## Results

### HOXA11-AS downregulation inhibits GC cells proliferation and induces cell cycle arrest

**Fig. 1:**
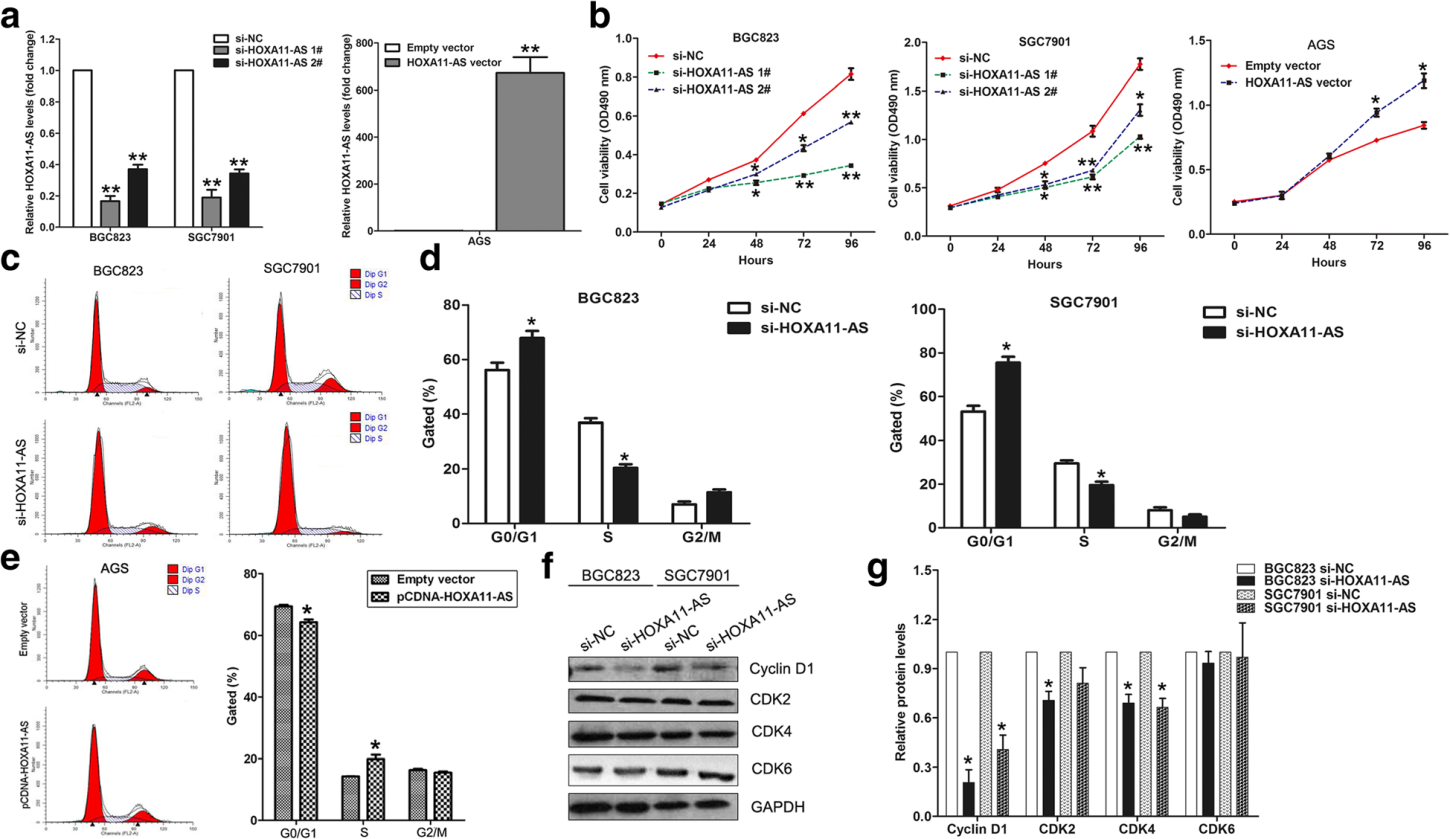
The effect of HOXA11-AS on gastric cancer cells proliferation and cell cycle progression. **a** qRT–PCR analysis of HOXA11-AS expression in BGC823, SGC7901 cells transfected with HOXA11-AS or NC siRNAs, and in AGS cells transfected with HOXA11-AS over-expression vector. **b** Growth curves of BGC823, SGC7901 and AGS cells after transfection with HOXA11-AS siRNAs or vector were determined by CCK8 assays. Values represented the mean ± s.e. from three independent experiments. **c**, **d** The cell cycle progression of BGC823 and SGC7901 cells was evaluated 48 h after transfection with HOXA11-AS siRNAs or NC using Flow cytometry assays. e The cell cycle progression of AGS cells was evaluated 48 h after transfection with HOXA11-AS vector or empty vector using Flow cytometry assays. The bar chart represented the percentage of cells in G0/G1, S, or G2/M phase, as indicated. **f**, **g** The cyclinD1, CDK2, CDK4 and CDK6 protein levels were detected in BGC823 and SGC7901 cells after transfection with HOXA11-AS siRNAs or NC using western blot. *P < 0.05, **P < 0.01

To determine the effect of HOXA11-AS on GC cells proliferation and cell cycle progression, two siRNAs was used to knockdown its expression and avoid off-target effect. As shown in Fig. 1a, these two siRNAs could significantly decrease HOXA11-AS in both BGC823 and SGC7901 cells. In addition, the qPCR results showed that HOXA11-AS expression was significantly upregulated after transfection with pcDNA-HOXA11-AS vector in AGS cells. Next, CCK8 assays showed that knockdown of HOXA11-AS impaired BGC823 and SGC7901 cells proliferation, while HOXA11-AS over-expression promoted AGS cells proliferation (Fig. 1b). To determine whether the effect of HOXA11-AS on GC cells growth reflected cell cycle arrest, cell cycle progression was analyzed by flow cytometry analysis. The results showed that BGC823 and SGC7901 cells transfected with si-HOXA11-AS had an obvious cell cycle arrest at the G1/G0 phase (Fig. 1c and d). Conversely, AGS cells transfected with HOXA11-AS vector had an decreased rate of G1 phase cells (Fig. 1e). Moreover, some cell cycle regulators levels were detected, and the results showed that the levels of Cyclin D1, and CDK2 were decreased in HOXA11-AS knockdown cells (Fig. 1f and g).

### Knockdown of HOXA11-AS inhibits GC cells migration and invasion

**Fig. 2:**
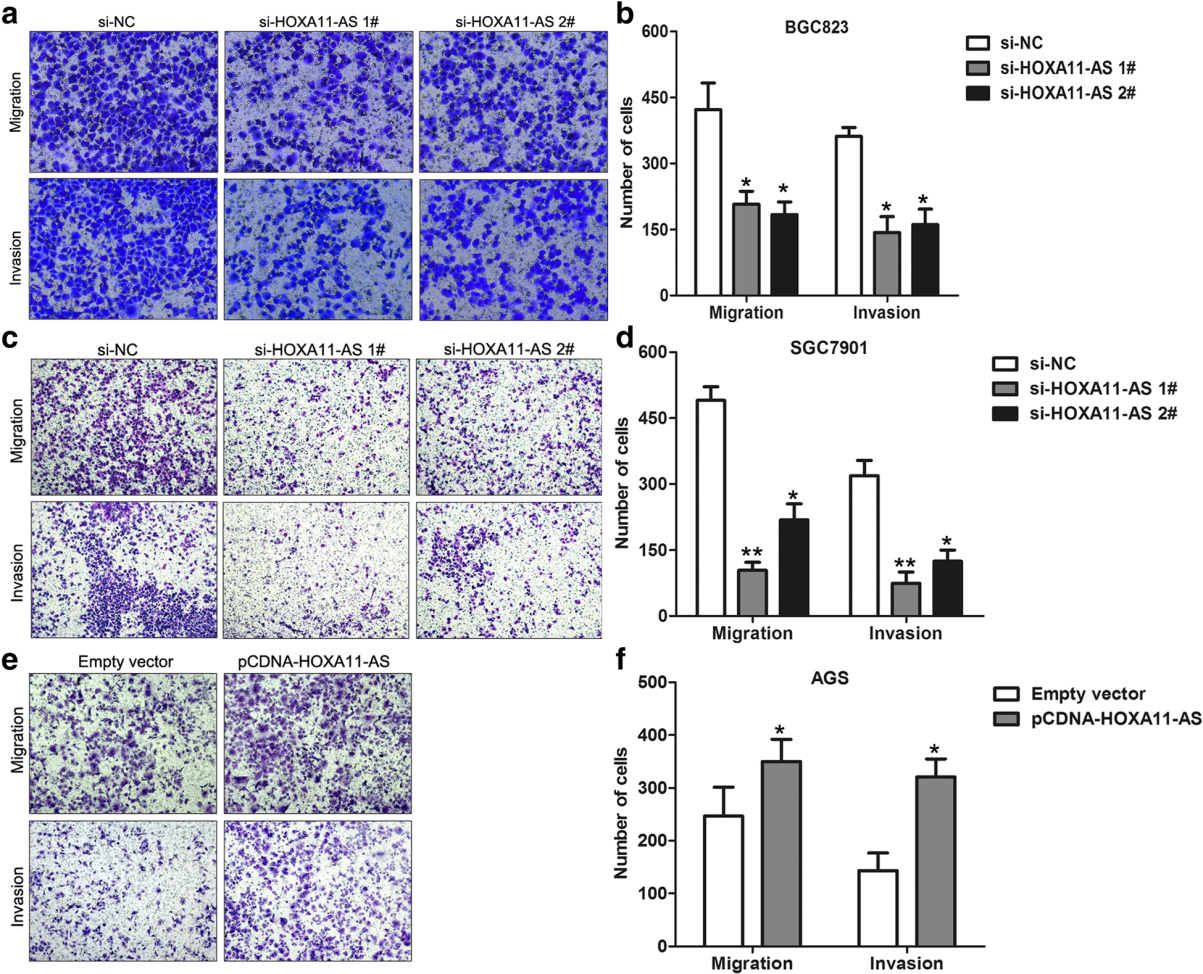
Knockdown of HOXA11-AS inhibits cell migration and invasion in GC. **a**, **b** The effect of HOXA11-AS knockdown on BGC823 cells migration and invasion was determined by transwell assays. **c**, **d** The effect of HOXA11-AS knockdown on SGC7901 cells migration and invasion was determined by transwell assays. **e**, **f** The effect of HOXA11-AS over-expression on AGS cells migration and invasion was determined by transwell assays. *P < 0.05, **P < 0.01

Tumor cells migration and invasion is a significant aspect of cancer progression. Here, we investigate the effect of HOXA11-AS on GC cells migration and invasive ability by performing transwell assays. The results showed that knockdown of HOXA11-AS expression impeded the BGC823 and SGC7901 cells migration and invasion compared with controls, while upregulation of HOXA11-AS promoted AGS cells migration and invasive ability (Fig. 2). Taken together, these data indicates that HOXA11-AS has important roles in GC progression.

### GC cells in vivo metastasis is impaired after HOXA11-AS knockdown

**Fig. 3:**
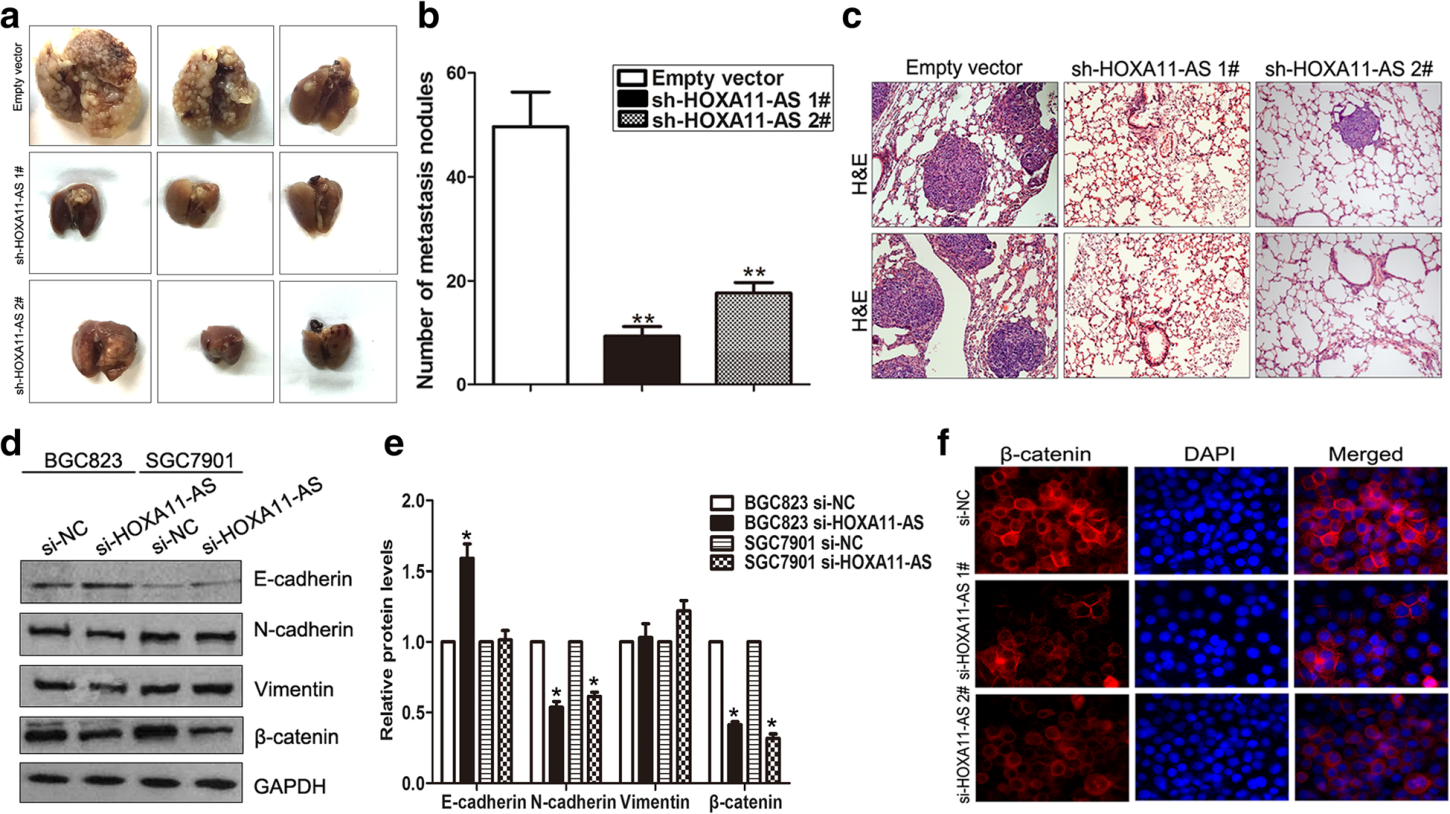
HOXA11-AS downregulation inhibits GC cells metastasis in vivo. **a** Analysis of an experimental metastasis animal model was performed by injecting HOXA11-AS stably downregulated BGC823 cells into nude mice. lungs from mice in each experimental group. **b** The numbers of metastais tumor nodules on lung surfaces were shown. **c** Visualization of the HE-stained lung sections. **d**, **e** The E-cadherin, N-cadherin, Vimentin and β-catenin protein levels were detected in BGC823 and SGC7901 cells after transfection with HOXA11-AS siRNAs or NC using western blot. **f** The β-catenin protein levels were detected in BGC823 cells after transfection with HOXA11-AS siRNAs or NC using Immunofluorescence.*P < 0.05, **P < 0.01

To confirm whether HOXA11-AS also affect GC cells metastasis in vivo, HOXA11-AS stably knockdown BGC823 cells and control cells were injected nude mice tail vein to determine whether HOXA11-AS would influence gastric cancer cells metastasis in vivo. 7 weeks after injection, the number of metastasis nodules in surface of lungs from HOXA11-AS knockdown group is less than that form control group (Fig. 3a and b). Moreover, H&E staining confirmed that knockdown of HOXA11-AS inhibited GC cells metastasis in vivo (Fig. 3c). Epithelial-Mesenchymal Transition plays important roles in cancer cells invasion and metastasis, and we found that lncRNA also involved in regulation of cancer cells EMT process in our previous studies. In this study, western blot assays showed that mesenchyma marker proteins including Vimentin and β-catenin levels were decreased in HOXA11-AS knockdown BGC823 cells, while epithelial marker E-cadherin levels had no significant change (Fig. 3d and e). In addition, Immunofluorescence analysis also showed that β-catenin protein levels were decreased in HOXA11-AS knockdown GC cells (Fig. 3f).

### HOXA11-AS interacts with EZH2, WDR5 and STAU1 in GC cells

**Fig. 4:**
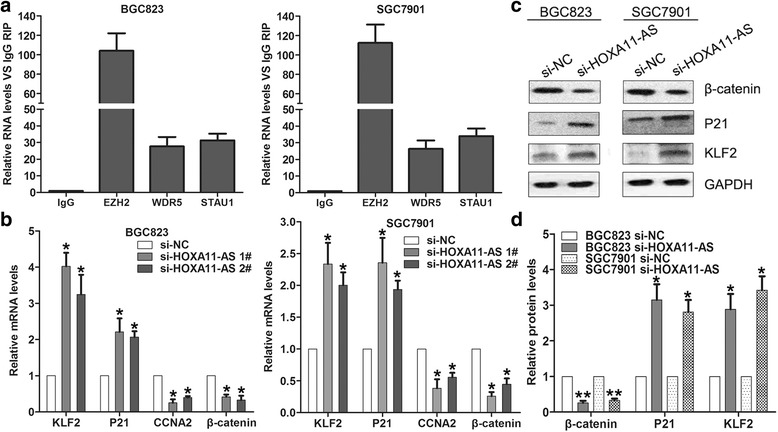
β-catenin, KLF2 and P21 are downstream targets of HOXA11-AS. **a** HOXA11-AS RNA levels in immunoprecipitates were determined by qRT-PCR. Expression levels of HOXA11-AS RNA were presented as fold enrichment relative to IgG immunoprecipitates. **b** qRT-PCR analysis of β-catenin, KLF2 and P21 expression in BGC823 and SGC7901 cells after transfection with HOXA11-AS or NC siRNA. **c**, **d** The β-catenin, KLF2 and P21 protein levels were detected in BGC823 and SGC7901 after transfection with HOXA11-AS siRNAs or NC using western blot. *P < 0.05, **P < 0.01

In our previous study, we found that HOXA11-AS could interact with several RNA binding proteins including EZH2, LSD1, DNMT1 et al. through RIP and RNA pulldown assays. Here, we also indicated that HOXA11-AS bind with WDR5 and STAU1 in GC cells by RIP analysis (Fig. 4a), which means HOXA11-AS could also regulate other underlying targets through different mechanisms. In addition to KLF2 and PRSS8, we also found some important cell cycle and metastasis regulators expression levels are altered in HOXA11-AS knockdown BGC823 cells through RNA sequencing, such as P21, CCNA2, and β-catenin. Further qPCR validation of the RNA sequencing results showed that KLF2 and P21 expression was increased in HOXA11-AS knockdown GC cells, while CCNA2 and β-catenin expression was decreased (Fig. 4b). Moreover, western blot analyses confirmed the qPCR results (Fig. 4c and d).

### HOXA11-AS bind with WDR5 and promotes β-catenin expression in GC cells

**Fig. 5:**
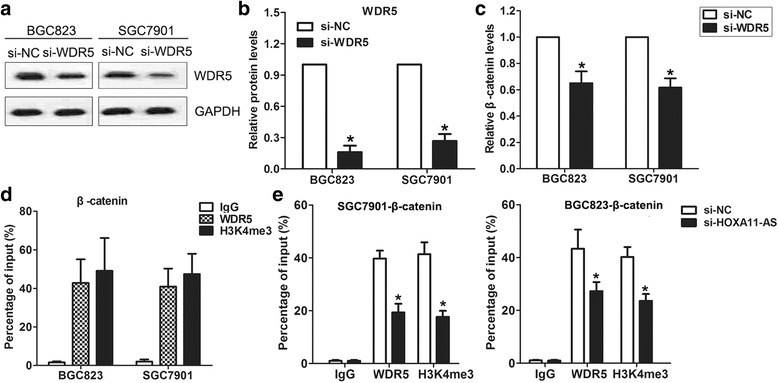
HOXA11-AS promotes β-catenin transcription by binding with WDR5. **a**, **b** Western blot analysis of WDR5 protein levels in BGC823 and SGC7901 cells after transfection with WDR5 or NC siRNA. **c** qRT-PCR analysis of β-catenin expression in BGC823 and SGC7901 cells after transfection with WDR5 or NC siRNA. **d** ChIP-qPCR analysis of WDR5, and H3K4me3 occupancy in the β-catenin promoter in BGC823 and SGC7901 cells. **e** ChIP-qPCR analysis of WDR5, and H3K4me3 occupancy in the β-catenin promoter in BGC823 and SGC7901 cells after transfection with HOXA11-AS or NC siRNA. *P < 0.05, **P < 0.01

Recent study demonstrated that WDR5 could be recruited by lncRNA and regulate its targets transcription [22], which suggests that HOXA11-AS may regulate β-catenin expression via interacting with WDR5. In order to verify our hypothesis, we firstly downregulated WDR5 expression by transfection with specific siRNA. As shown in Fig. 5a and b, the WDR5 protein levels were decreased after transfection with siRNA. Meanwhile, qPCR analysis indicated that β-catenin expression was also decreased by ~40% (Fig. 5c). Furthermore, ChIP assays showed that WDR5 could directly bind to β-catenin promoter regions, however, knockdown of HOXA11-AS reduced WDR5′s binding to β-catenin promoter (Fig. 5d and e). These data indicate that HOXA11-AS regulates β-catenin, at least, partly through interacting with WDR5 in GC cells.

### HOXA11-AS regulates KLF2 and P21 expression by interacting with STAU1 and EZH2

**Fig. 6:**
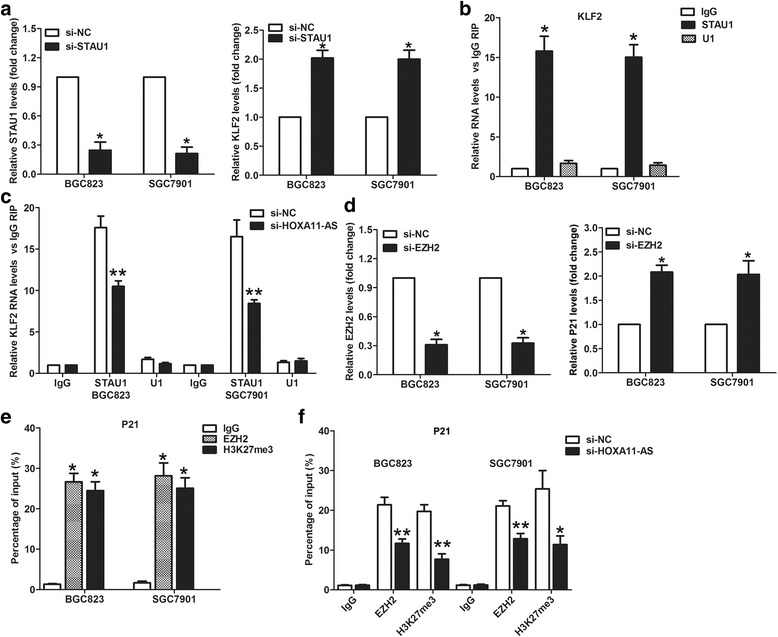
HOXA11-AS represses P21 transcription and KLF2 expression by binding with EZH2 and STAU1. **a** qRT-PCR analysis of STAU1 and KLF2 expression in BGC823 and SGC7901 cells after transfection with STAU1 or NC siRNA. **b** ChIP-qPCR analysis of EZH2, and H3K27me3 occupancy in the P21 promoter in BGC823 and SGC7901 cells. **c** ChIP-qPCR analysis of EZH2, and H3K27me3 occupancy in the P21 promoter in BGC823 and SGC7901 cells after transfection with HOXA11-AS or NC siRNA. **d** qRT-PCR analysis of EZH2 and P21 expression in BGC823 and SGC7901 cells after transfection with EZH2 or NC siRNA. **e** KLF2 mRNA levels in immunoprecipitates were determined by qRT-PCR. Expression levels of KLF2 mRNA were presented as fold enrichment relative to IgG immunoprecipitates. **f** KLF2 mRNA levels in immunoprecipitates were determined by qRT-PCR after transfection with HOXA11-AS or NC siRNA. *P < 0.05, **P < 0.01

In our previous study, we found that HOXA11-AS repressed KLF2 expression at transcriptional level through interacting with EZH2 and DNMT1. Interestingly, RIP results showed that HOXA11-AS could also bind with SATU1, which also can interact with lncRNA and promote KLF2 mRNA degradation. Importantly, qPCR analyses showed that KLF2 expression was increased by 2-folds after knockdown of STAU1 by siRNA in GC cells (Fig. 6a). Moreover, RIP assays determined that STAU1 could bind with KLF2 mRNA, and knockdown of HOXA11-AS reduced their binding in GC cells (Fig. 6b and c). In addition, we found that EZH2 downregulation could increase P21 expression in GC cells (Fig. 6d), which is consistent with knockdown of HOXA11-AS. Further ChIP analysis revealed that EZH2 could bind to P21 promoter region and knockdown of HOXA11-AS reduced EZH2 binding efficiency (Fig. 6e and f). These findings indicate that HOXA11-AS could regulate KLF2 and P21 expression through other mechanisms except which we have documented in our previous study.

## Discussion

Recent studies have highlighted lncRNAs as critical players in tumorigenesis and cancer progression processes; however, the potential functions and underlying mechanistic details of these lncRNAs in GC still remain unclear. In our previous study, we performed comprehensive analysis of aberrantly expressed lncRNAs in GC using TCGA stomach cancer and normal tissue RNA sequencing data and four independent microarray data sets from Gene Expression Omnibus. As a result, we indentified an GC associated lncRNA HOXA11-AS, which is significantly overexpressed in GC tissues and promotes proliferation and invasion of GC cells by scaffolding the chromatin modification factors PRC2, LSD1 and DNMT1 [21]. In this study, we further found that upregulated HOXA11-AS also promoted GC cells cell cycle progression and in vivo metastasis. These findings suggest that HOXA11-AS exerts oncogenic function in GC, and its overexpression contributes to GC tumorigenesis and progression.

In addition to our studies, Wang et al. reported that HOXA11-AS expression was closely related with glioma patients poor prognosis, and could be an independent prognostic factor for glioblastoma multiforme patients [23]. Another study by Zhang et al. revealed that HOXA11-AS was highly expressed in both lung adenocarcinoma and squamous cell carcinoma tissues [24]. However, some studies indicated that HOXA11-AS also have tumor suppressor function in other cancers. For instance, Li et al reported that HOXA11-AS expression was decreased in the colorectal cancer tissues and cells, and significantly associated with CRC patients poor prognosis and carcinoembryonic antigen level [25]. Besides, HOXA11-AS expression was significantly down-regulated in human EOC epithelial ovarian cancer, and an exonic variant (rs17427875, A > T) within HOXA11-AS exerts tumor suppressive function [26]. These findings suggest that HOXA11-AS has tissue specific expression pattern and could functions as either oncogenes or tumor suppressors depending on the circumstance.

Although the expression pattern and clinical relevance of HOXA11-AS in several human cancers has been reported, the underlying mechanisms for HOXA11-AS in these cancers remain unclear. In this study, mechanistic investigation showed that HOXA11-AS could promote β-catenin expression through binding with WDR5, induce KLF2 mRNA degradation via interacting with STAU1, and recruit EZH2 to P21 promoter and repress P21 transcription. β-catenin is the key regulator of Wnt/β-catenin signaling pathway, which is usually aberrantly activated in human cancers and contribute to tumor initiation and cancer progression [27–29]. Activation of this signaling pathway is also involved in gastric cancer progression through regulation of Epithelial-mesenchymal transition [30]. KLF2 is a member of Kruppel-like factor (KLF) family, which contains Cys2/His2 zinc-finger domains and acts as transcriptional repressors or activators to regulate multiple gene transcription [31]. Recent studies revealed that KLF2 expression is diminished in several human cancers and exhibits tumor-suppressor features for its inhibitory effect on cell proliferation and induction of cell apoptosis [32, 33]. Our previous study also showed that KLF2 are downregulated in GC, and KLF2 overexpression impaired cell growth and invasion [21].

## Conclusions

In summary, our study showed that over-expressed lncRNA HOXA11-AS promotes GC cell proliferation, cell cycle progression and metastasis in vivo, suggesting that it exhibits oncogenic properties in GC tumorigenesis and progression. HOXA11-AS exerts its oncogenic effects partially through activation of β-catenin transcription, epigenetic silencing of P21 expression, and inducing KLF2 mRNA degradation via interacting with WDR5, EZH2 and STAU1. Our findings further the understanding of GC pathogenesis, and facilitate the development of lncRNA-directed diagnostics and therapeutics against this disease.

## Additional file

Additional file 1: Primers and siRNAs sequences. (XLSX 13 kb)

## Abbreviations

ChIP: Chromatin immunoprecipitation
EZH2: Enhancer of zeste homolog 2
GC: Gastric cancer
H3K27me3: Histone H3 lysine 27 trimethylation
lncRNAs: Long noncoding RNAs
ncRNAs: Noncoding RNAs
RIP: RNA immunoprecipitation
